# Blood Pressure Control in Smokers with Arterial Hypertension Who Switched to Electronic Cigarettes

**DOI:** 10.3390/ijerph13111123

**Published:** 2016-11-11

**Authors:** Riccardo Polosa, Jaymin B. Morjaria, Pasquale Caponnetto, Eliana Battaglia, Cristina Russo, Claudio Ciampi, George Adams, Cosimo M. Bruno

**Affiliations:** 1 UOC di Medicina Interna e d’Urgenza Edificio 4, Piano 3, AOU “Policlinico-V. Emanuele”, University of Catania, Via S. Sofia 78, 95123 Catania, Italy; p.caponnetto@unict.it (P.C.); eliana.battaglia@hotmail.it (E.B.); cmbruno@unict.it (C.M.B.); 2 Centro per la Prevenzione e Cura del Tabagismo (CPCT), “Policlinico-V. Emanuele”, University of Catania, 95123 Catania, Italy; 3 Internal and Emergency Medicine, “Policlinico-V. Emanuele”, University of Catania, 95123 Catania, Italy; 4 Department of Respiratory Medicine, Royal Brompton & Harefield Hospital NHS Trust, Harefield Hospital, Hill End Road, Harefield UB9 6JH, UK; jbmorjaria@gmail.com; 5 MCAU ARNAS Garibaldi, 95100 Catania, Italy; kristina_russo@yahoo.com (C.R.); claudiociampi@yahoo.it (C.C.); 6 Rex Hospital, UNC Health System, Raleigh, NC 27607, USA; george.adams@unchealth.unc.edu

**Keywords:** smoking cessation, electronic cigarette, blood pressure, hypertension, tobacco harm reduction

## Abstract

Electronic cigarettes (ECs) are battery-operated devices designed to vaporise nicotine, which may help smokers with quitting or reducing their tobacco consumption. No data is available regarding the health effects of ECs use among smokers with arterial hypertension and whether regular use results in blood pressure (BP) changes. We investigated long-term changes in resting BP and level of BP control in hypertensive smokers who quit or reduced substantially their tobacco consumption by switching to ECs. A medical records review of patients with hypertension was conducted to identify patients reporting regular daily use of ECs on at least two consecutive follow-up visits. Regularly smoking hypertensive patients were included as a reference group. A marked reduction in cigarette consumption was observed in ECs users (*n* = 43) though consumption remained unchanged in the control group (*n* = 46). Compared to baseline, at 12 months (follow-up visit 2) decline in cigarette consumption was associated with significant reductions in median (25th-, 75th-centile) systolic BP (140 (134.5, 144) to 130 (123.5, 138.5) mmHg; *p* < 0.001) and diastolic BP (86 (78, 90) to 80 (74.5, 90) mmHg; *p* = 0.006). No significant changes were observed in the control group. As expected, decline in cigarette consumption in the ECs users was also associated with improved BP control. The study concludes that regular ECs use may aid smokers with arterial hypertension reduce or abstain from cigarette smoking, with only trivial post-cessation weight gain. This resulted in improvements in systolic and diastolic BP as well as better BP control.

## 1. Introduction

Smoking is the leading cause of preventable premature mortality in the world. Death is mainly caused by ischemic heart disease, stroke, lung cancer, and the catastrophic complications of advanced stage chronic obstructive pulmonary disease [1]. It has been estimated that the 10-year fatal cardiovascular risk is doubled in smokers [2], and that for young smokers the risk for myocardial infarction is up to 5-fold higher compared to non-smokers [2,3]. The risk is primarily related to the amount of tobacco smoked daily and shows a clear dose-response relationship with no obvious lower limit for deleterious effects [4,5]. By sustaining low-grade systemic inflammation [6] and contributing to arterial stiffness [7], tobacco smoke is also likely to lead to arterial hypertension [8], thus further worsening smokers’ cardiovascular risk profile.

The interaction between smoking and blood pressure (BP) is complex. Despite the strong relationship between smoking and elevated risk for cardiovascular disease [9,10], there is paucity and discrepancy about the long-term effects of smoking cessation on BP in already established hypertension [11,12,13].

Electronic cigarettes (ECs) are battery-operated devices designed to vaporise nicotine without burning tobacco. These consumer products share many similarities with smoking in the behavioural aspect of their use [14]. Users are predominantly smokers, who report using them long-term as an alternative for conventional cigarettes, to reduce cigarette consumption or quit smoking, to relieve tobacco withdrawal symptoms, and to continue having a “smoking” experience [15], but with much reduced health risks [16,17].

Data from clinical trials of “healthy” smokers [18,19,20] and in vulnerable populations [21,22] have shown that ECs may help smokers with quitting or reducing their tobacco consumption and their use is well tolerated. There is no data about changes in smoking behaviour after daily ECs use among smokers with arterial hypertension. Most importantly, it is unknown if long-term smoking cessation/reduction after switching to regular “vaping” (the act of inhaling vapour from ECs) in patients taking anti-hypertensive medications could result in improved resting BP and better BP control.

Here we report, for the first time, long-term improvement in resting BP as well as in level of BP control in smokers with a diagnosis of hypertension who quit or reduced substantially their tobacco consumption by switching to ECs.

## 2. Methods

### 2.1. Patient Population

We conducted a medical records review of patients with arterial hypertension regularly followed-up at the outpatient clinics of our institution. Patients reporting regular daily use of ECs on at least two consecutive follow-up visits were eligible to be included in our study. Data from a second group of age-, sex-matched patients who reported to be regular smokers on at least two consecutive follow-up visits was included as a reference group. Patients in both study groups had to have similar weight (<5 kg) and systolic BP (<10 mmHg) fluctuations between pre-baseline and baseline visits. Any patients with a known cause of secondary hypertension were excluded. The study was approved by the local institutional ethics review board (Protocol registered No. 675).

### 2.2. Study Design

From chart review, we extracted patient data from the clinic visit immediately preceding [baseline visit] the first of the two consecutive follow-up visits (follow-up visit 1 and 2). We also included data from the clinic visit immediately prior to the baseline visit [pre-baseline visit] to substantiate disease stability. In brief, data from four visits were collected and analysed. Pre-baseline visits were carried out at 6–12 months prior to baseline visits. Follow-up visit 1 and 2 were carried out at 6 (±1) and 12 (±2) months after baseline visits, respectively. At each routine outpatient clinic visit patients were assessed using a standard approach consisting of clinical examination, review of smoking history, and measurement of blood pressure (BP), heart rate (HR), and body weight. Systolic (SBP) and diastolic BP (DBP) measurements were conducted according to the recommendations of the Seventh Report of the Joint National Committee on Prevention, Detection, Evaluation, and Treatment of High Blood Pressure [23] with patients seated for at least 5 min prior to measurements. Two BP measurements, spaced 1–2 min apart, were obtained by auscultation using a mercury sphygmomanometer and an appropriately sized cuff. The average of the two readings was used for analysis. Body weight was measured by using a mechanical column scale with patients removing shoes and heavy clothing.

### 2.3. Data Management

For the purposes of this study, patients’ data at the time of the outpatient visits were later extracted from their medical record and entered into an electronic spreadsheet prior to statistical computation.

### 2.4. Approach to Treatment

Our routine approach to anti-hypertensive treatment is to evaluate and optimize patients’ drug regimens to include a combination of drugs that work on different pathophysiological pathways in order to maximize BP control [24]. Where appropriate diuretics, vasodilators, sympatholytics, and renin-angiotensin-aldosterone system blockers are prescribed. Changes to medications were made after review of both BP office measurements and BP home readings and were recorded. Self-reported adherence to medications was assessed at each visit.

### 2.5. Study Outcomes

The primary outcome of interest was the change in resting SBP and DBP from baseline to the final follow-up visit at about one year. Secondary outcomes of interest were changes from baseline to the final follow-up visit in: (a) proportion of blood pressure grading; (b) proportion of patients with good BP control, defined as an office SBP < 140 mmHg and a DBP < 90 mmHg (<130/80 mmHg for patients with diabetes, chronic kidney disease, or cardiovascular disease) [24]; (c) HR; (d) body weight; and (e) the number, class, and dosage of medications. We also collected information about safety and tolerability of EC use in the patients using them.

### 2.6. Analyses

Data were expressed as mean (±standard deviation (SD)) if parametric and median (interquartile range (IQR)) if non-parametric. We also delineated data for single (ECs use only) and dual users (combined ECs and conventional cigarettes use). Statistical comparisons of change from baseline between and within the two groups in the parameters assessed were carried out using student’s *t*-test and Wilcoxon-signed rank test depending on whether the data was parametric or not, respectively. Similar statistical analyses were conducted for dual and single users within groups from baseline. Missing measurements were not included in the analyses. Repeated measures analysis of variance (ANOVA) between baseline and 12 months and between the two study groups were also conducted. A two-tailed *p* value of less than 0.05 was considered to indicate statistical significance. Odds ratios (OR) were computed to compare changes in BP control. All analyses were performed with the Statistical Package for Social Science (SPSS for windows version 18.0, Chicago, IL, USA). 

## 3. Results

### 3.1. Patients’ Characteristics

A total of 89 (50 male, 39 females) regular smokers with a diagnosis of hypertension and on anti-hypertensive drugs at baseline were studied. They had either pre-hypertension, or grade 1 or 2 hypertension according to 2013 ESH/ESC criteria [24] despite taking at least three antihypertensive agents (by and large a mixed combination of thiazide diuretics, angiotensin-converting enzyme inhibitors, angiotensin II receptor blockers, calcium channel blockers, or β-blockers). Forty-three patients (26 male, 17 females) reported regular daily use of ECs at two consecutive follow-ups. ECs use ranged from 10 to 14 months, with 36/43 (83.7%) patients using them for more than a year. Among the EC user group 28 out of 43 reported previous quit attempts (only three attempts were medically assisted); 31 out of 46 in the reference group reported previous quit attempts (only four attempts were medically assisted). There were no significant differences in all study measures between the pre-baseline and baseline visits. Pre-baseline and baseline patient demographics and characteristics are summarised in Table 1.

### 3.2. Changes in Smoking Behaviour and Patterns of E-Cigarette Use

A marked reduction in conventional cigarette consumption was observed in regular daily ECs users, their mean (±SD) cigarettes/day use decreasing from 20.2 (±5.0) at baseline to 2.6 (±2.9) at follow-up visit 1 and to 1.8 (±2.0) at follow-up visit 2, respectively (*p* < 0.001 for both visits) (Table 2). As expected, no significant reduction in conventional cigarette consumption was observed in the reference group. Dual usage was reported by 23/43 (53.5%) patients at follow-up visit 1 and 22/43 (51.2%) at follow-up visit 2, respectively (Table 3). A significant reduction in conventional cigarette consumption was also observed in dual users, with their mean (±SD) cigarettes/day use decreasing from 21.5 (±6.9) at baseline to 4.8 (±2.3) at follow-up visit 1 and to 3.7 (±1.1) at follow-up visit 2, respectively (*p* < 0.001 for both visits) (Table 3). More than a 75% reduction from baseline in cigarettes/day consumption was reported by 14/23 (60.9%) dual users at follow-up visit 1 and by 17/22 (77.3%) at follow-up visit 2, respectively.

### 3.3. Changes in BP, HR, and Body Weight

**Figure 1:**
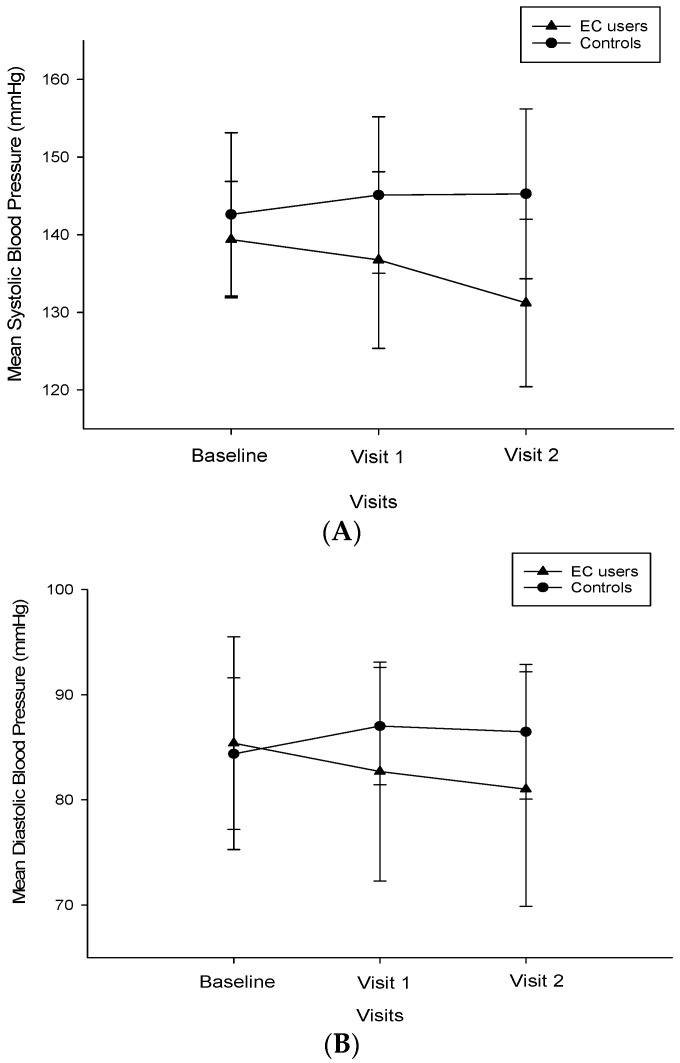
(**A**) Changes in systolic blood pressure from baseline, at visit 1 (6 ± 1 months) and visit 2 (12 ± 2 months) separately for electronic cigarettes users (closed triangles) and controls (closed circles). All data expressed as mean and error bars are standard error of the mean; (**B**) Changes in diastolic blood pressure from baseline, at visit 1 (6 ± 1 months) and visit 2 (12 ± 2 months) separately for electronic cigarettes users (closed triangles) and controls (closed circles). All data expressed as mean and error bars are standard error of the mean. Abbreviations: mmHg, millimeters of mercury.

**Figure 2:**
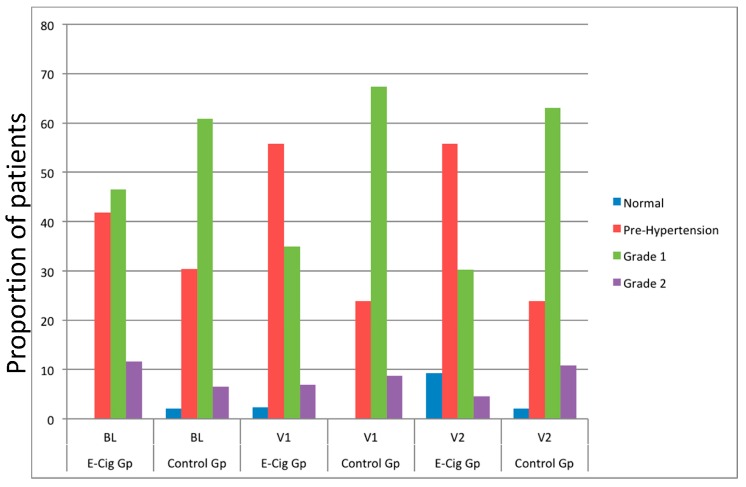
Proportion of blood pressure grading at baseline, visit 1 (6 ± 1 months) and visit 2 (12 ± 2 months) separately for electronic cigarettes users and controls. (Normal, blue; Pre-Hypertension, red; Grade 1, green; Grade 2, purple). Abbreviations: BL—baseline; V1—Follow-up visit 1; V2—Follow-up visit 2.

Changes in SBP and DBP from baseline in smokers with arterial hypertension who switched to ECs and between the two study groups are illustrated and reported in Figure 1A,B and Table 2. A significant reduction in median SBP (*p* < 0.001) and DBP (*p* = 0.006) from baseline was observed at follow-up visit 2 in the EC group. In contrast, no significant change in BP was observed in the reference group. The observed reductions in SBP and DBP were significant (*p* < 0.001, both for SBP and DBP) when comparing the EC group to the reference group at 12 months. Table 3 illustrates changes in health outcomes separately for exclusive EC users and dual users; subgroup analyses show that with the exception of reduced cigarette consumption and SBP at 12 months—no significant changes from baseline were observed in dual users. The overall proportion of patients with grades 1 and 2 hypertension at baseline (58.1%) was much less at follow-up visit 1 (41.9%) and 2 (34.9%) in the EC group compared to the reference group, where the proportion of grade 1 and 2 hypertension at baseline (67.4%) increased at both follow-up visits (Visit 1: 76.1%; Visit 2: 73.9%). This was reciprocated by an increase in the proportion of patients with normal and pre-hypertension for the former and by a reduction in the latter group respectively (Figure 2).

No significant change in HR from baseline was noted in both patients’ groups during the course of the study (Table 2). A significant, but small, increase in body weight was observed in both study groups at the 2nd follow-up visit (Table 2); however, the observed small weight gain was not significant between the EC group and the reference group. Table 3 illustrates changes in mean body weight separately for exclusive EC users and dual users from baseline; subgroup analysis shows a small but significant increase in body weight in exclusive EC users (from 71.0 kg to 74.2 kg), whereas no significant increase is observed in dual users (from 75.6 kg to 76.7 kg).

### 3.4. Changes in BP Control and Anti-Hypertensive Medications

**Figure 3:**
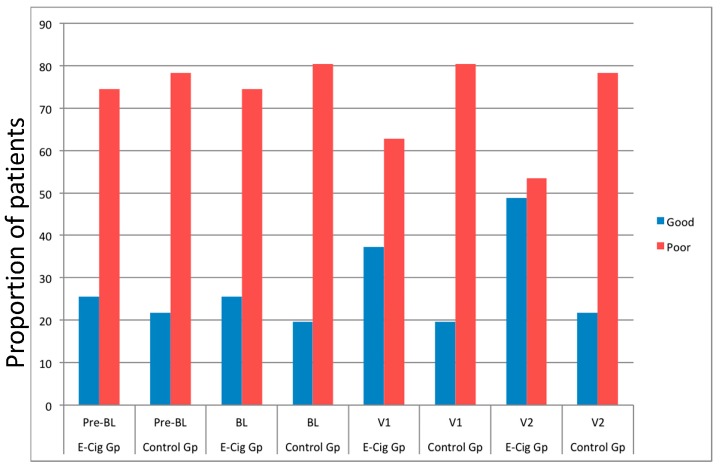
Proportion of good (blue bar) and poor (red bar) patients’ blood pressure control at baseline, at visit 1 (6 ± 1 months), and visit 2 (12 ± 2 months) separately for electronic cigarettes users and controls. Abbreviations: Pre-BL—Pre-baseline; BL—baseline; V1—Follow-up visit 1; V2—Follow-up visit 2.

The overall proportion of patients in the EC group with good BP control at baseline increased from 25.6% to 37.2% and 48.8% at follow-up visit 1 and 2, respectively (Figure 3; Table 4). In contrast, the proportion of patients in the reference group with good BP control at baseline (19.6%) was virtually unchanged during the course of the study (Visit 1: 19.6%; Visit 2: 21.7%). The OR of improved BP control in the EC group compared to the controls at Visit 1 and 2 was 2.29 and 5.29, respectively.

Overall, all patients remained on a stable number of anti-hypertensives throughout the study period with minor adjustments in medications’ class and dosage (Table 5). 

### 3.5. Safety and Tolerability

No severe adverse reactions or acute decompensation in BP were reported with EC use throughout the entire follow-up duration. Overall, EC use appeared to be well tolerated in these hypertensive patients with dry mouth and throat irritation being occasionally reported.

## 4. Discussion

Smoking is known to act synergistically to elevate the risk of myocardial infarction in patients with established hypertension [25]. Moreover, there is emerging evidence that arterial hypertension may be one of the many recognized deleterious effects of tobacco smoking [26]. For these reasons, quitting smoking is among the most important steps patients with elevated BP can take to improve their cardiovascular health [5]. Abstinence has been the preferred strategy to address these harmful effects, but has been largely unsuccessful with modest quit rates being reported [27,28].

Here we illustrate for the first time the utility of ECs as a bridge in reducing cigarette consumption in smokers with arterial hypertension. Adoption of regular long-term ECs use in this group led to a marked and stable reduction in conventional cigarette consumption, with about 50% of them abstaining completely from tobacco smoking by the end of the observation period. The success rate observed in these patients with arterial hypertension is similar to that recently reported in asthmatics [22] and may be explained by the notion that ECs are a valid long-term alternative nicotine source to conventional cigarettes due to their many similarities to smoking behaviour [14,29]. Moreover, ECs use was well tolerated with no reported severe adverse reactions or acute decompensation in BP.

As a result of the substantial reduction in cigarette consumption, decreased systolic and diastolic BP as well as improved BP control was also reported. In the EC group, systolic and diastolic BP fell by 10 mmHg and 6 mmHg, respectively. Improvement in systolic (but not diastolic) BP was also observed in dual users at 12 (but not 6) months. These findings are in agreement with the 8.8 mmHg reduction in systolic BP at week-52 in a prospective randomised control trial looking at the effect of smoking cessation by using ECs in subjects with high BP at baseline [30]. Predictably, not only was there a decrease in the proportion of patients with hypertension, but also the overall proportion of patients in the EC group with good BP control increased five-fold compared to the reference group. Of note, improvement in resting BP as well as in level of BP control was unrelated to the minor adjustments in the use of anti-hypertensives.

Given the well-established acute effect of smoking on immediate vasopressor and tachycardic responses [31,32] and increased arterial stiffness [33], the observed reduction in BP after long-lasting smoking reduction or abstinence is not surprising. Nonetheless, in view of the complex interaction between smoking and blood pressure (BP) and the reported discrepancy about the long-term effects of smoking cessation on BP in already established hypertension [11,12,13], our positive findings require explanation. Besides important methodological limitations of population studies that may predispose them to heterogeneous results [26], the association of elevated risk for future development of hypertension after smoking cessation [13] has been mainly attributed to post-cessation weight gain [8,34]. Low BP in smokers is related to decreased body weight [35] with higher body weight and elevated BP being more common in former smokers compared to non-smokers [36]. Thus, when interpreting BP variations in the context of smoking abstinence, post cessation weight gain becomes a critical confounder because the negative effects of weight gain on BP could outweigh the positive effects of smoking cessation. In the current study, we have observed that quitters who use ECs seem to limit substantially their post-cessation weight gain; a significant but small, increase in body weight (i.e., 1.2 kg) was observed at the second follow-up visit, but the observed small weight gain was not significant between EC group and reference group (1.2 kg vs. 0.7 kg). The trivial post-cessation weight gain after switching to regular EC use might have contributed to the positive long-term effects of smoking cessation on BP and BP control. Moreover, improved weight control has been shown in a recent one-year randomized smoking cessation trial of smokers who quit smoking by switching to ECs [37]. By alleviating weight gain, EC use may ultimately deter the hypertensive comorbidities of both smoking and obesity. Additionally, it must be noted that we investigated a relatively “young” population with a short duration of hypertension history and probably less established vascular remodelling; this might also have contributed to the positive effects on BP and BP control.

In agreement with the findings from other research groups [38,39], positive improvements in BP after smoking cessation were noted not only in quitters, but also in those who reduced consumption in conventional cigarettes (i.e., dual users). A possible explanation is that dual users in our study substantially reduced their average cigarettes/day consumption, with 77.3% reporting a reduction of at least 75% from baseline by the end of the observation period. Of note, that reduction in tobacco smoking thanks to ECs may have led to improvements in BP which is also consistent with the results of a recent prospective RCT [30]. The multiple linear regression model in which the SBP change from BL to week 52 was entered as a dependent variable and tested against continuous smoking phenotype classification, sex, age, and weight change as independent variables, demonstrated that the mean reduction in systolic BP from baseline at one-year remained significantly associated with both smoking reduction and smoking abstinence, with the b coefficient for quitters being more than two-fold greater compared to reducers.

The observed improvement in systolic and diastolic BP as well as in BP control in hypertensive smokers who switched to regular ECs use suggests that the harmful effects of cigarette smoke on the vascular system can potentially be reversed. By substantially reducing exposure to conventional cigarettes’ hazardous toxicants and achieving clinically relevant BP reductions, EC use may not only improve the cardiovascular risk profile but also confer an overall health advantage in smokers unable or unwilling to quit who are also at risk of developing arterial hypertension compared to continuing smoking. The use of low risk nicotine-containing products (including ECs) should be investigated as a safer alternative approach to harm-reversal (i.e., specific reversal of BP elevation) and, in general, to harm-reduction (i.e., overall reduction of cardiovascular risk associated with tobacco smoking).

There are some limitations in our study. First, the sample size is small; hence the results must be interpreted with caution. However, despite being small, significant results were reported for several study endpoints. Standard concerns associated with retrospective studies (including variance in the quality of information recorded by medical professionals and difficulty in establishing a causal relationship) need consideration. Nonetheless, a clear advantage conducting this type of study is the generation of hypotheses that can be tested prospectively under controlled conditions. Secondly, patients in this study may represent a self-selected sample, which is not representative of all smokers with arterial hypertension (e.g., smokers not intending to quit, switching to ECs). Thirdly, although careful extraction from patients’ medical records allowed systematic assessment of anti-hypertensive medication prescriptions, this alone may not reflect true usage due to lack of rigorous adherence checks. Carbon monoxide measurements are not routinely performed in patients who are followed-up for elevated blood pressure at outpatient clinics and their smoking abstinence was self-reported. However, self-reported number of cigarettes smoked per day in studies of this type is not subjected to the kind of biases observed in clinical trials where there is the tendency to claim abstinence [40]. Moreover, similar beneficial effects were also reported in dual users (i.e., smoking reducers) and therefore objective measures of abstinence are unlikely to be of great importance. Given the design of this study, it was impossible to provide details about specific products and patterns of use, but this (lack of) knowledge will not change study findings or their interpretation. Last but not least, lifestyle changes (e.g., salt intake, diet, recreational exercise), which may have an influence on BP measures, were not taken into account. Nonetheless, by including a reference group, which was carefully matched for age, sex, weight, and systolic BP fluctuations between pre-baseline and baseline visits, we minimized the observation bias.

## 5. Conclusions

That ECs may help also smokers with arterial hypertension to reduce their cigarette consumption or remain abstinent is a novel and important message of the study. As a result of this, we have observed substantial long-term improvements in systolic and diastolic BP as well as greater BP control. Large prospective studies are now warranted to confirm and expand the potential role of the e-vapor category for smoking cessation and/or reversal of harm in hypertensive patients who smoke. Substitution of conventional cigarettes with EC is unlikely to raise significant health concerns and may achieve better BP control. This can improve counseling between physicians and their patients with high BP using or intending to use ECs [41].

## Figures and Tables

**Table 1:** Baseline measurements in all subjects prior to initiation of electronic cigarette and control group.

Parameter	Pre-Baseline	Baseline	*p* Value between Pre-Baseline and Baseline	*p* Value between Groups at Baseline
**E-Cigarette Group (*n* = 43)**				
Gender	26 M, 17 F	-	-	-
Age (years) ^§^	53.5 (±6.3)	-	-	0.623
Smoking pack years ^§^	37.4 (±14.0)	-	-	0.718
FTND ^§^	5.6 (±2.2)	-	-	0.49
Weight (Kg) ^§^	73.0 (±12.2)	73.4 (±12.2)	0.053	0.942
Conventional Cigarettes/day ^§^	21.5 (±6.9)	20.2 (±5)	0.143	0.274
SBP (mmHg)	142 (134, 145.5)	140 (134.5, 144)	0.754	0.1
DBP (mmHg)	86 (79.5, 91.5)	86 (78, 90)	0.338	0.563
Hypertension stage ^#^				
Normal	0	0	-	-
Pre-hypertension	16	18	-	-
Stage I	21	20	-	-
Stage II	6	5	-	-
HR (bpm)	80 (67.5, 88)	77 (69.5, 84.5)	0.588	0.911
**Control Group (*n* = 46)**				
Gender	24 M, 22 F	-	-	
Age (years) ^§^	54.2 (±7.5)	-	-	
Smoking pack years ^§^	38.4 (±11.6)	-	-	
FTND ^§^	5.9 (±2.0)	-	-	
Weight (Kg) ^§^	72.9 (±12.4)	73.2 (±12.4)	0.217	
Conventional Cigarettes/day ^§^	21.5 (±5.8)	20.5 (±5.2)	0.096	
SBP (mmHg)	143 (135.8, 149.3)	143 (135, 151.8)	0.553	
DBP (mmHg)	85.5 (80, 90)	85 (80, 90)	0.239	
Hypertension stage ^#^				
Normal	0	1		
Pre-hypertension	15	14		
Stage I	28	28		
Stage II	3	3		
HR (bpm)	80 (69, 84.8)	77.5 (72, 85)	0.709	

Abbreviations: M, male; F, female; Kg, kilogram; mmHg, millimetres of mercury; bpm, beats per minute; FTND, Fagerstrom test for nicotine dependence. ^#^ Hypertension stages based on American Hypertension Association guidelines [24]; ^§^ data expressed as mean (±SD); Data expressed as median (25th-, 75th-centile).

**Table 2:** Parameter measurements at baseline, 6- and 12-months for both groups.

Parameter	Baseline (BL)	6 Months		12 Months		*p* Value between Groups Baseline to 12 Months ^ψ^
E-Cigarette Group (*n* = 43; 26 M, 17 F)			*p* Value ^¥^		*p* Value ^§^	
Weight (Kg) ^¶^	73.4 (±12.2)	75.1 (±13.7)	0.012	74.6 (±13.5)	0.046	0.455
Cigarettes/day ^¶^	20.2 (±5.0)	2.6 (±2.9)	<0.001	1.8 (±2.0)	<0.001	<0.001
SBP (mmHg)	140 (134.5, 144)	137 (130, 142.5)	0.141	130 (123.5, 138.5)	<0.001	<0.001
DBP (mmHg)	86 (78, 90)	81 (75, 89)	0.053	80 (74.5, 90)	0.006	<0.001
HR (bpm)	77 (69.5, 84.5)	78 (71, 86)	0.414	78 (72.5, 89.5)	0.745	0.705
**Control Group (*n* = 46; 24 M, 22 F)**			***p* Value ^¥^**		***p* Value ^§^**	
Weight (Kg) ^¶^	73.2 (±12.4)	73.8 (±12.4)	0.074	73.9 (±12.6)	0.043	
Cigarettes/day ^¶^	20.5 (±5.2)	20.8 (±5.5)	0.532	21.4 (±5.6)	0.223	
SBP (mmHg)	143 (135, 151.8)	145 (137.3, 151.8)	0.105	145 (136.3, 150)	0.095	
DBP (mmHg)	85 (80, 90)	87 (85, 90)	0.007	85 (85, 90)	0.042	
HR (bpm)	77.5 (72, 85)	78 (72, 83.5)	0.791	78.5 (72, 83.8)	0.857	

Abbreviations: *n*, number; M, male; F, female; Kg, kilograms; mmHg, millimetres of mercury; bpm, beats per minute. Data expressed as median (25th-, 75th-centile); SBP, systolic blood pressure; DBP, diastolic blood pressure; HR, heart rate. ^¶^ Data expressed as mean (±standard deviation); ^¥^ *p* value computed between baseline and 6 months; ^§^ *p* value computed between baseline and 12 months; ^Ψ^ *p* value computed as repeated measures ANOVA between baseline and 12 months between groups.

**Table 3:** Parameters measurements at baseline, 6- and 12-months for the E-Cigarette group only.

Parameter	Baseline	1st Follow-up Visit (6 Months ± 1)		2nd Follow-up Visit (12 Months ± 2)	
	**All Patients (*n* = 43; 26 M, 17 F)**				
Weight (Kg) ^¶^	73.4 (±12.2)	75.1 (±13.7)		74.6 (±13.5)	
Conventional Cigarettes/day ^¶^	20.2 (±6.9)	2.6 (±2.9)		1.8 (±2.0)	
SBP (mmHg)	140 (134.5, 144)	137 (130, 142.5)		130 (123.5, 138.5)	
DBP (mmHg)	86 (78, 90)	81 (75, 89)		80 (74.5, 90)	
HR (bpm)	77 (69.5, 84.5)	78 (71, 86)		78 (72.5, 89.5)	
	**Single Users**				
		***n* = 20 (11 M; 9 F)**		***n* = 21 (11 M; 10 F)**	
			***p* Value ^§^**		***p* Value ^Ω^**
Weight (Kg) ^¶^	71.0 (±11.8)	75.0 (±14.6)	<0.001	74.2 (±14.8)	0.003
Conventional Cigarettes/day ^¶^	20.4 (±4.5)	-	-	-	
SBP (mmHg)	140 (135, 146.3)	134 (130, 142.3)	0.010	130 (123, 138)	<0.001
DBP (mmHg)	85.6 (75.8, 92)	80.5 (73.8, 84.8)	0.019	80 (75, 87)	0.030
HR (bpm)	76 (71.5, 81.3)	79.5 (75, 86)	0.523	80 (76, 90)	0.151
	**Dual Users**				
		***n* = 23 (15 M; 8 F)**		***n* = 22 (15 M; 7 F)**	
			***p* Value ^§^**		***p* Value ^Ω^**
Weight (Kg) ^¶^	75.6 (±12.4)	75.2 (±13.1)	0.544	76.7 (±11.3)	0.382
Conventional Cigarettes/day ^¶^	20.1 (±5.4)	4.8 (±2.3)	<0.001	3.7 (±1.1)	<0.001
SBP (mmHg)	138 (133.5, 140.5)	137 (132, 143.5)	0.750	130 (120.8, 139.8)	0.011
DBP (mmHg)	87 (78.5, 90)	83 (80, 91.5)	0.691	80 (70.8, 90)	0.109
HR (bpm)	83 (68.5, 86)	77 (70, 83)	0.095	76 (70.5, 92.3)	0.874

Abbreviations: *n*, number; M, male; F, female; Kg, kilograms; mmHg, millimetres of mercury; bpm, beats per minute. Data expressed as median (25th-, 75th-centile). ^¶^ Data expressed as mean (±standard deviation); ^§^ *p* value computed between baseline and 6 months within group; ^Ω^ *p* value computed between baseline and 12 months within group.

**Table 4:** Changes in Hypertension Control over time in the E-Cigarette (*n* = 43) and Control Groups (*n* = 46).

Visits	Pre-Baseline		Baseline		Visit 1		Visit 2	
Hypertension Control	E-Cig. Group (%)	Control Group (%)	E-Cig. Group (%)	Control Group (%)	E-Cig. Group (%)	Control Group (%)	E-Cig. Group (%)	Control Group (%)
Patients with BP Good Control	11	10	11	9	16	9	21	10
(<140/90 mmHg in subjects with no co-morbidities; <130/80 mmHg in subjects with co-morbidities)	(25.6)	(21.7)	(25.6)	(19.6)	(37.2)	(19.6)	(48.8)	(21.7)
Patients with BP Poor Control	32	36	32	37	27	37	22	36
(≥140/90 mmHg in subjects with no co-morbidities; ≥130/80 mmHg in subjects with co-morbidities)	(74.4)	(78.3)	(74.4)	(80.4)	(62.8)	(80.4)	(51.2)	(78.3)

**Table 5:** Details of anti-hypertensive medications use at baseline, 6- and 12-months for both study groups.

Parameter	Pre-Baseline	Baseline	6 Months	12 Months
**E-Cigarette Group (*n* = 43; 26 M, 17 F)**				
Number of drugs ^¶^	3.7 (±1.3)	3.6 (±1.1)	3.5 (±1.3)	3.3 (±1.5)
Drugs’ class (%)				
*- Diuretics*	*75%*	*78%*	*75%*	*72%*
*- Calcium channel blockers*	*43%*	*41%*	*33%*	*30%*
*- Beta-blockers*	*31%*	*28%*	*25%*	*23%*
*- ACE inhibitors*	*90%*	*92%*	*90%*	*90%*
*- Angiotensin receptor blockers*	*75%*	*75%*	*73%*	*73%*
Change of drugs’ class from baseline (%)	-	-	5 (11.6%)	5 (11.6%)
Same dose as from baseline (%)	-	-	35 (81.4%)	33 (76.7%)
Reduction in dose from baseline (%)	-	-	5 (11.6%)	7 (16.3%)
Increase in dose from baseline (%)	-	-	3 (7.0%)	3 (7.0%)
**Control Group (*n* = 46; 24 M, 22 F)**				
Number of drugs ^¶^	3.5 (±1.3)	3.7 (±1.4)	3.5 (±1.4)	3.6 (±1.3)
Drugs’ class (%)				
*- Diuretics*	*74%*	*72%*	*70%*	*74%*
*- Calcium channel blockers*	*40%*	*37%*	*35%*	*31%*
*- Beta-blockers*	*27%*	*25%*	*25%*	*26%*
*- ACE inhibitors*	*88%*	*90%*	*92%*	*93%*
*- Angiotensin receptor blockers*	*80%*	*82%*	*83%*	*86%*
Change of drugs’ class from baseline (%)	-	-	8 (17.4%)	9 (19.6%)
Same dose as from baseline (%)	-	-	37 (80.4%)	35 (76.1%)
Reduction in dose from baseline (%)	-	-	4 (8.7%)	5 (10.9%)
Increase in dose from baseline (%)	-	-	5 (10.9%)	6 (13.0%)

Abbreviations: *n*, number; M, male; F, female; ACE – angiotensin converting enzyme. ^¶^ Data expressed as mean (±standard deviation).
